# The Immunobiology of Dry Eye Disease: A Review of the Pathogenesis, Regulation and Therapeutic Implications

**DOI:** 10.3390/ijms262110583

**Published:** 2025-10-30

**Authors:** Sarah Jacqueline Saram, Maya Natasha Thomas, Leo Feinberg, Harry W. Roberts, Conor M. Ramsden, Małgorzata Woronkowicz, Piotr Skopiński

**Affiliations:** 1 Ministry of Health, 16 College Road College of Medicine Building and Singapore, Singapore 169854, Singapore; sarah.saram@mohh.com.sg; 2 North Devon District Hospital, Royal Devon University Healthcare NHS Foundation Trust, Barnstaple EX31 4JB, UK; mayanatasha.thomas@nhs.net (M.N.T.); malgorzata.woronkowicz.14@alumni.ucl.ac.uk (M.W.); 3 West of England Eye Unit, Royal Devon University Healthcare NHS Foundation Trust, Exeter EX2 5DW, UK; leo.feinberg@nhs.net (L.F.); conor.ramsden@nhs.net (C.M.R.); 4 Faculty of Health and Life Science, University of Exeter Medical School, Exeter EX1 2HZ, UK; 5 Moorfields Eye Hospital NHS Foundation Trust, London EC1V 2PD, UK; 6 Department of Ophthalmology, SPKSO Ophthalmic University Hospital, Medical University of Warsaw, 00-576 Warsaw, Poland; 7 Department of Histology and Embryology, Medical University of Warsaw, 02-004 Warsaw, Poland

**Keywords:** dry eye disease, ocular surface, immunopathogenesis, innate immunity, adaptive immunity, T cells, tear biomarkers

## Abstract

Dry eye disease (DED) is increasingly recognized as a condition driven by immune dysregulation at the ocular surface (OS). Chronic inflammation, mediated by aberrant activation of both innate and adaptive immune pathways, underlies disease progression and symptom persistence. Neuroimmune interactions further amplify OS inflammation, contributing to epithelial damage and impaired homeostatic regulation. This review summarizes current literature on the immunopathogenesis of DED, highlighting the complex interplay of molecular mechanisms of innate and adaptive immune activation, neuroimmune-mediated inflammation, and emerging molecular and cellular biomarkers. In addition, we examine existing and emerging therapeutic strategies that target these immune-molecular pathways, including precision immunomodulatory approaches, to inform future management of DED. By integrating mechanistic insights with clinical findings, this review aims to provide a comprehensive overview of the molecular mechanisms underlying the dysregulated immune response associated with DED.

## 1. Introduction

Dry eye disease (DED) is a prevalent and multifactorial disorder impacting the ocular surface (OS), and is characterized by tear film instability, hyperosmolarity, and inflammation, affecting approximately 10–20% of people over the age of 40 globally [1,2]. Once primarily viewed as a disorder of tear deficiency or excessive evaporation, this perspective has evolved to recognize the significant role of the loss of immune homeostasis and dysregulation of the innate and adaptive immune system in driving disease progression and symptom persistence [2,3].

Over the past decade, advances in immunology, molecular profiling, and animal models have reshaped our understanding of DED pathogenesis. Accumulating evidence has elucidated complex immunoregulatory dysfunction at the OS, including aberrant activation of epithelial stress pathways, innate immune triggers, T cell polarization, and impaired regulatory networks [2,4,5,6]. A better understanding of the molecular mechanisms underlying DED has facilitated the identification of potential precision immunomodulatory techniques to mitigate disease progression.

This review underscores the central role of immune dysregulation in DED, reframing it as a complex disorder of disrupted immune homeostasis rather than just a binary deficiency–evaporation paradigm. We discuss evidence from human and preclinical models implicating aberrant innate and adaptive immune responses and neuroimmune interactions as key drivers of disease initiation and persistence, and highlight how these insights are shaping the development of emerging targeted immunomodulatory therapies.

## 2. Methodology

In this narrative review, a comprehensive search strategy was developed to identify relevant publications focusing on the role of the immune system in the pathogenesis of DED. Literature searches were performed using electronic databases, including PubMed, Google Scholar, Web of Science, and Scopus, covering the period from January 2000 to July 2025. The following combinations of Medical Subject Headings (MeSH) terms and keywords were used: *“dry eye disease,” “dry eye syndrome,” “ocular surface immunology,” “innate immunity,” “adaptive immunity,” “T cells,” “dendritic cells,” “cytokines,” “chemokines,” “tear biomarkers,”* and *“immunopathogenesis.”* To ensure comprehensive coverage of the literature, the reference lists of the included articles were screened to identify further relevant publications. No limitations were placed on study type, but we excluded those that did not focus specifically on immune dysregulation at the OS.

## 3. OS Immune Homeostasis

**Figure 1:**
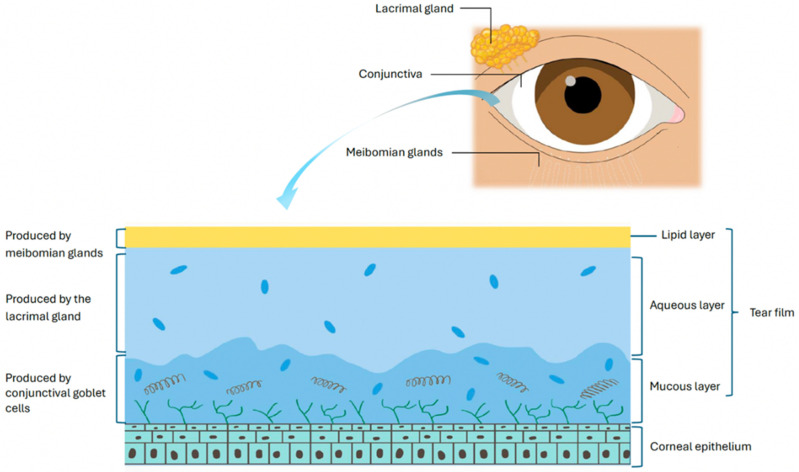
The components of the tear film.

The OS in healthy eyes is actively kept in a strict immune homeostasis by various regulatory mechanisms that, when disrupted, can lead to uncontrolled inflammation seen in DED [3,7]. The tear film components, produced by the lacrimal glands, meibomian glands, and OS epithelia, act as an immunologically active barrier that preserves corneal clarity through lubrication, suppresses local inflammation, and promotes wound healing [8,9] (Figure 1). It contains a diverse array of growth factors, antimicrobial peptides, and antibodies, including immunoglobulin A (IgA) and G (IgG), which help to defend the OS from microorganisms and maintain homeostasis [9,10].

The lacrimal gland produces the aqueous component of the tear film along with antimicrobial proteins, immunoglobulins, and cytokines that regulate lubrication, epithelial integrity, and immune tolerance [11,12,13,14,15,16,17].

Complementing the aqueous layer, the meibomian glands supply the lipid layer that prevents tear evaporation. The meibomian glands have immune-regulatory functions, helping maintain OS homeostasis by controlling lipid secretion and limiting microbial overgrowth. Dysregulation of this immune balance, particularly through pathogenic Th17 responses and IL-17A–driven neutrophil recruitment, contributes to glandular obstruction, inflammation, and dysfunction [18].

The cornea has a unique immune-privileged status maintained through several mechanisms. Its avascular and alymphatic structure prevents the infiltration of circulating leukocytes and migration of antigen presenting cells (APCs) to regional lymphoid tissues, thereby dampening the adaptive immune response [3,5]. Furthermore, the cornea lacks mature resident APCs, which express low levels of major histocompatibility complex class II (MHC II) and lack co-stimulatory molecules such as CD80, CD86, and CD40, further contributing to corneal immunosenescence [3,19].

The corneal epithelium serves as a physical barrier (with glycocalyx and tight junctions) against microorganisms and environmental insults. It actively participates in immunoregulation by expressing immunomodulatory molecules such as programmed death ligand 1 (PD-L1), which promote apoptosis of active effector T cells [3,20]. Corneal and conjunctival epithelial cells express functional toll-like (TLR) and NOD-like receptors (NLR), enabling pathogen detection and recognition of danger-associated molecular patterns (DAMPs) to initiate appropriate immune responses [20].

The cornea is a highly innervated surface and relies on neuropeptides derived from nerves such as substance P to help with immunoregulation by regulating virus and bacteria-induced inflammation and maintaining corneal epithelial homeostasis [3,21]. However, oversecretion of neuropeptides seen in DED can lead to pathological amplification of the immune response and propagation of the ‘vicious cycle’ of inflammation [3].

## 4. Definition and Classification of DED

The classification of DED has evolved to aid diagnostic precision and guide targeted therapy by identifying the suspected etiology of the disease [22]. Originally divided into tear-deficient and evaporative types, this was refined in 2007 to distinguish between aqueous-deficient dry eye (ADDE) and evaporative dry eye (EDE), with ADDE often further subclassified into Sjögren’s syndrome (SS) and non-Sjögren’s etiologies [5,23,24]. ADDE is characterized by insufficient or reduced tear volume, whilst EDE is due to over-evaporation of the tear film despite normal tear production. However, it is recognized that many patients exhibit overlapping features of both subtypes, with co-existing disturbance in tear quantity and quality, underscoring the complex and multifactorial nature of DED [2,22].

## 5. Pathophysiology of DED

### 5.1. The Role of Tear Hyperosmolarity in DED

**Figure 2:**
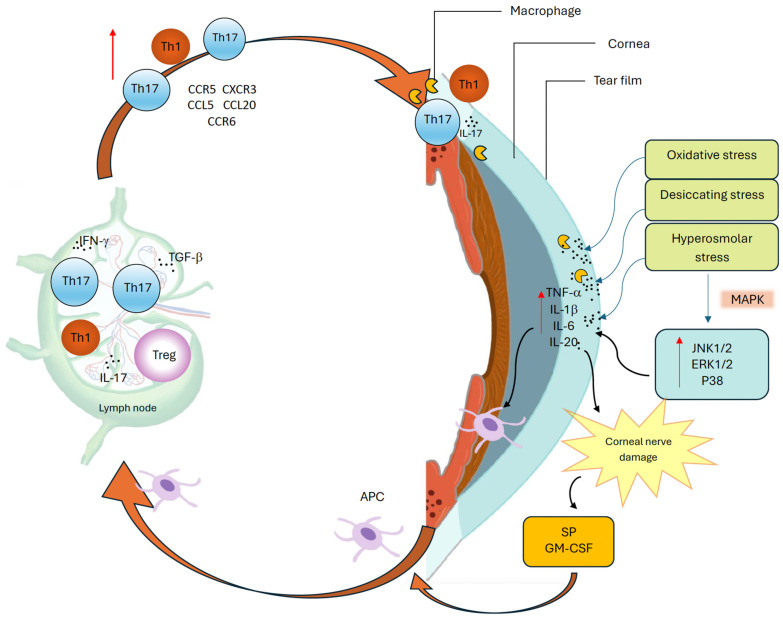
The vicious cycle of dry eye disease.

Tear hyperosmolarity has been identified as a key feature of DED and underlies both reduced aqueous production and increased evaporation. It may represent the initiating insult, driving a cascade of OS damage wherein inflammation and immune dysregulation establish a dynamic, self-sustaining cycle that disrupts normal homeostatic control (Figure 2) [2,4]. This hyperosmolar state activates an epithelial stress response, causing the release of proinflammatory cytokines and matrix metalloproteinases (MMP), which promote marked recruitment and activation of both innate and adaptive immune cells to the OS [2,25]. Type 1 helper (Th1) and type 17 helper (Th17) lymphocytes are the predominant T cell subtypes in DED and cause IFN-γ and IL-17–mediated epithelial damage [5,26]. This immune-driven disruption of the homeostasis of the OS further destabilizes the tear film, increases evaporation, and perpetuates the ‘vicious cycle’ that characterizes chronic DED [22,26]. Other extrinsic (e.g., contact lens wear, LASIK surgery, use of systemic anti-cholinergics) and intrinsic factors (e.g., age, female sex, autoimmune conditions such as SS) can contribute to the cycle and further the chronicity of the disease [26]. While this ‘vicious cycle’ is dynamic and multifactorial, with overlap between innate and adaptive immune responses, for simplicity and clarity’s sake, the authors felt it important to break down each structure in turn, while reflecting the complex interplay between the two.

This cycle is driven by oxidative, desiccating, and hyperosmolar stress at the ocular surface, which activates mitogen-activated protein kinase (MAPK) pathways, including c-Jun N-terminal kinase 1/2 (JNK1/2), extracellular signal-regulated kinase 1/2 (ERK1/2), and p38 [27,28], and induces proinflammatory mediators, such as tumor necrosis factor-alpha (TNF-α), interleukin-1 beta (IL-1β), interleukin-6 (IL-6), and interleukin-20 (IL-20) [27,29]. These cytokines promote antigen-presenting cell (APC) maturation and migration via lymphatics to regional lymph nodes, where effector T helper 1 (Th1) and T helper 17 (Th17) cells are activated. Dry eye disease (DED)-primed Th1 cells expressing C-C chemokine receptor type 5 (CCR5) and C-X-C chemokine receptor type 3 (CXCR3), and Th17 cells expressing CCR6, are recruited back to the ocular surface (OS) through corresponding chemokine gradients, including C-C motif chemokine ligand 5 (CCL5) and C-C motif chemokine ligand 20 (CCL20) [30]. Interferon-gamma (IFN-γ) enhances APC maturation, while transforming growth factor-beta (TGF-β) promotes Th17 differentiation and weakens immune regulation [22,26]. At the OS, IL-1β and TNF-α amplify inflammation and barrier disruption, IL-20 enhances macrophage recruitment and signaling, and interleukin-17 (IL-17) induces matrix metalloproteinase (MMP) production, epithelial damage, and apoptosis. Neuroimmune dysregulation further amplifies this cycle: proinflammatory cytokines damage peripheral nerves, triggering substance P (SP) and granulocyte-macrophage colony-stimulating factor (GM-CSF) release from sensory nerve endings, which enhances major histocompatibility complex class II (MHC-II) expression and promotes T cell priming, further exacerbating ocular surface inflammation [31]. Together, these immune and neuroimmune interactions perpetuate chronic inflammation and sustain the vicious cycle of DED.

### 5.2. Aging and Immune Dysregulation in DED

Aging is a major risk factor for DED, driving both immune dysregulation and structural changes across the OS [32]. Aged C57BL/6 mice serve as a particularly informative model, developing meibomian gland dysfunction, corneal staining, GC depletion, and lymphocytic infiltration of the conjunctiva and lacrimal glands [33,34]. Aging promotes the generation of autoreactive CD4^+^ T cells that infiltrate ocular tissues, contributing to chronic inflammation and goblet cell loss [35]. While DC density and morphology remain largely unchanged, age-related immune dysregulation may instead reflect functional alterations in APCs. IFN-γ plays a central role in this process, mediating GC depletion, while aged regulatory T cells (Tregs) lose their suppressive function and instead secrete IFN-γ and IL-17, further amplifying inflammation [36,37,38]. Furthermore, aged mice models demonstrate higher levels of memory Th-17 cells compared with their younger counterparts, which correlate with disease severity upon re-exposure to desiccating stress [39].

### 5.3. The Role of the Microbiome in DED

Whilst the OS is a site of low bacterial load, both preclinical and clinical studies suggest that the ocular and gut microbiome are interlinked in sustaining immune tolerance and preventing OS inflammation in DED [40]. In CD25KO mice (lacking CD25, the α-chain of the IL-2 receptor, resulting in Treg deficiency), absence of commensal bacteria at the OS amplified autoreactive CD4^+^IFN-γ^+^ T-cell responses and accelerated SS-like dacryoadenitis, whereas fecal transplantation restored immune regulation, highlighting a key microbiome–T cell axis in DED pathogenesis [41]. Complementary evidence from de Paiva and colleagues showed in murine models exposed to antibiotics and desiccating stress that loss of *Firmicutes/Bacteroidetes* and expansion of *Proteobacteria* promotes pathogenic CD4^+^ effector T-cell responses, diminishes regulatory T-cell and IL-13–mediated goblet cell support, and exacerbates OS inflammation [42].

Clinical studies further support a link between microbiome dysbiosis and immune dysregulation in DED. Liang et al. reported heterogeneous conjunctival dysbiosis in DED patients, with depletion of commensals, reduced α-diversity, and overabundance of specific bacterial and fungal species (e.g., Staphylococcus aureus, Malassezia globosa), potentially disrupting mucosal tolerance and OS immune homeostasis [43].

In the gut, dysbiosis is also prevalent in SS and correlates with OS disease severity, characterized by loss of protective taxa such as *Bifidobacterium* and *Actinobacteria* and enrichment of *Prevotella* and *Veillonella*. These microbial shifts are thought to be mechanistically linked to altered short-chain fatty acid (butyrate) production, impaired epithelial barrier integrity, and skewed Th1/Th17 versus Treg balance, suggesting a gut–eye axis role in DED [44]. Additionally, enrichment of proinflammatory taxa such as *Corynebacterium tuberculostearicum* and *Propionibacteriaceae* has been observed at the OS in DED with postulated activation of NF-κB/TLR2 signaling and ROS-mediated cytokine release, further driving OS inflammation [45].

Overall, whilst these findings underscore the potential immune regulatory role of commensal microbiota in maintaining homeostasis at the OS, mechanistic studies remain limited, and small cohort sizes constrain generalizability.

## 6. Innate Immune System in DED

In response to stressors such as tear hyperosmolarity, the innate immune system at the OS mounts a rapid, non-specific defense [46]. In DED, however, this protective response can become maladaptive, triggering proinflammatory signaling cascades, promoting an influx of immune cells, and priming the adaptive immune response, thus impairing immune homeostasis at the OS [6,47,48,49].

### 6.1. Epithelial Cells’ Stress Response and the Inflammatory Cascade

Stressed corneal epithelial cells release a rapid and high concentration of proinflammatory cytokines, which contribute to local inflammation [6,27]. Direct hyperosmolar stress to these cells rapidly activates mitogen-activated protein kinases (MAPKs), causing sustained activation of their three signaling pathways: c-Jun N-terminal kinase 1/2 (JNK1/2), extracellular signal-regulated kinase 1/2 (ERK1/2), and p38 [27,28]. These pathways transduce extracellular signals to intracellular responses, which upregulate inflammation, apoptosis, and immune response through the expression of cytokines and MMPs [50,51].

MMP-9, a key proteolytic enzyme upregulated by proinflammatory cytokines IL-1β and TNF-α, contributes to OS damage by degrading epithelial basement membrane components and disrupting tight junction proteins such as Zonula Occludens-1 (ZO-1) and Occludin, thereby weakening corneal barrier integrity in DED [27,50,52]. Moreover, increased nuclear transcription factors like Nuclear Factor kappa-light-chain-enhancer of activated B cells (NF-κB) and Nuclear Factor of Activated T cells 5 (NFAT5), drive the early upregulation of cytokines such as TNF-α, IL-1β, IL-6, and IL-20, creating a microenvironment conducive to immune cell activation and recruitment [27,29].

Wang et al. demonstrated, using multiple murine models of DED, alongside analyses of human tear samples, that IL-20 is upregulated and may act pathogenically by promoting macrophage recruitment, epithelial apoptosis, and Th17 responses [29]. While this study provides important translational insight by linking IL-20 elevation to human disease, the mechanistic evidence is largely derived from animal and in vitro models.

Further T cell recruitment to the OS is promoted by C-C motif chemokine ligand 5 (CCL5), C-X-C motif chemokine ligand 9 (CXCL9), CXCL10 chemokines, as well as Th17-inducing cytokines such as IL-6, TGF-β, IL-23, and IL-17A [6,37]. Recent work by Liu et al. demonstrated through murine DED models that basal epithelial cells, particularly fibroblast-like subtypes generated via epithelial–mesenchymal transition, can act as ‘non-professional APCs’ at the OS. These cells upregulate MHC class II and secrete chemokines such as CCL2, promoting macrophage recruitment and activating adaptive immune responses, thereby linking innate and adaptive immunity. This proinflammatory microenvironment persists in the chronic phase, with enduring fibrotic changes in epithelial cells and polarization of macrophages toward an inflammatory subtype, contributing to sustained immune-driven inflammation in DED [53]. However, these findings are again based solely on murine models, and whether the same immunopathological mechanisms occur in chronic human DED remains uncertain.

Toll-like receptor 4 (TLR4) is expressed on various corneal and conjunctival epithelial cells and requires co-receptors CD14 and myeloid differentiation factor 2 (MD2) to detect bacterial lipopolysaccharide (LPS) [46]. Under normal conditions, oversignaling is regulated through low TLR4 expression on apical cells and the absence of MD2, which prevents unnecessary immune responses to commensal bacteria [46]. Redfern et al. observed reduced TLR 4 and 9 protein expression in human corneal epithelial cells in vitro when exposed to dry eye conditions, whilst TLR 5 levels were increased or unchanged [54]. The same group also reported elevated levels of DAMPs, such as high mobility group box 1 (HMGB1) and heat shock protein (HSP-60), in the tear films of DED patients, which are associated with activation of the innate immune response via TLRs (especially TLR4) and increased cytokine and MMP-9 production, perpetuating OS inflammation [55]. This apparent discrepancy, low receptor protein yet high ongoing DAMP-driven activation, may be a reflection that in vitro findings may not fully capture the dynamics of in vivo chronic human DED pathogenesis or indicate that the mechanism of TLR dysregulation is not yet fully understood.

### 6.2. The Complement System

The complement system, a central arm of the innate immune defense, has been increasingly implicated in the pathogenesis of DED. Several clinical studies have shown that both the classical and alternative complement pathways and their regulatory proteins are present and functionally active in the normal eye, including the cornea, aqueous humor, and tears [56,57,58,59]. In murine eyes, complement was found to be constitutively active at a low basal level, with C3 cleavage fragments and terminal complex deposition detectable in ocular tissues, providing continuous immune surveillance. This activity is tightly regulated by complement regulatory proteins, where inhibition of decay-accelerating factor (DAF) or membrane cofactor protein (MCP) precipitated severe intraocular inflammation. Regulation at the level of C3 convertase has been identified as a critical checkpoint in preventing complement-driven injury [60]. Human studies corroborate these findings, showing that complement regulatory proteins, including DAF, MCP, CD59, factor H, and factor I, are widely distributed throughout ocular tissues, while aqueous and vitreous humor exhibit inhibitory activity against both the classical and alternative pathways in vitro [57,61,62,63,64].

In DED, clinical studies have demonstrated that tear fluid from DED patients with meibomian gland dysfunction contains elevated levels of complement activation products C3a and C5a compared with controls, indicating local complement activation and implicating innate immune dysregulation, particularly in elderly patients [65]. Preclinical models further support a mechanistic role for complement: DED-specific autoantibodies trigger OS inflammation in a complement-dependent manner, resulting in neutrophil infiltration, proinflammatory cytokine release, and GC loss. Complement depletion attenuates these effects, highlighting its function as a mediator linking humoral and T cell–driven immune responses in DED [66]. However, evidence for complement involvement in DED remains limited, mostly from small cohorts or murine models, whereas it is better characterized in other ocular diseases such as age-related macular degeneration [67].

### 6.3. Innate Immune Cells

#### 6.3.1. Neutrophils

The increased release and production of proinflammatory cytokines and chemokines in DED causes abnormal immune cell infiltration and functioning [27,47]. Neutrophils contribute to inflammation not only through phagocytosis and reactive oxygen species (ROS) production but also by releasing neutrophil extracellular traps (NETs) in response to tear hyperosmolarity [47,68]. These NETs can form in the absence of infection, perpetuating sterile inflammation and promoting chronic OS damage through sustained cytokine expression and type I IFN signaling [68,69]. Kwon et al. identified the presence of anti-citrullinated protein autoantibodies (ACPA) in the OS wash of 40% of patients with DED, likely generated through peptidylarginine deiminase 4 (PAD4)-mediated citrullination of proteins during neutrophil NETosis. These ACPAs were found to subsequently induce OS inflammation in murine models [70].

#### 6.3.2. Macrophages

Macrophages are versatile innate immune cells involved in pathogen clearance, tissue repair, and immune regulation. Differing activation states exist, including Classically activated (M1) proinflammatory macrophages and alternatively activated (M2) anti-inflammatory macrophages, depending on their environment [47]. In DED, hyperosmolar stress skews macrophage polarization toward a proinflammatory M1 state while suppressing anti-inflammatory M2 activity, promoting Th1 and Th17 infiltration, and amplifying OS immune dysregulation.

A recent study by Alam et al. using single-cell RNA sequencing in murine models of DED showed a three-fold increase in resident macrophages. Moreover, the phenotypic shift in macrophages towards pain sensitization via gene expression of CXCL1 and loss of homeostatic gene expression was observed. Increased production of neurosensitizing factors such as CXCL1 can activate transient receptor potential vanilloid 1 (TRPV-1), A disintegrin, and metalloproteinase 17 (ADAM17), contributing to ocular pain and epithelial barrier disruption associated with DED [71]. Complementing these findings, recent single-cell transcriptomic and epigenomic profiling has revealed distinct conjunctival macrophage subsets in DED mouse models, including a regulatory retinoid X receptor alpha (RXRα), a nuclear receptor modulating immune gene expression that suppresses inflammation via IL-10 signaling, the depletion of which exacerbates goblet cell loss and Th17-mediated pathology [72]. This highlights the capacity of macrophages to serve as a conduit between innate and adaptive immunity, where hyperosmolarity-driven M1 polarization not only amplifies local inflammation but also orchestrates T cell recruitment and activation, thereby propagating immune dysregulation in DED.

#### 6.3.3. Natural Killer (NK) Cells

NK cells play a pivotal role in OS immunity by secreting large amounts of IFN-γ, which activates surrounding macrophages and T cells. In addition to their immunomodulatory function, NK cells exhibit cytotoxic activity via granzyme and perforin release, contributing to tissue damage [47]. The role of NK cells in the early stages of DED pathogenesis has been suggested, as their activation promotes IFN-γ–mediated inflammation and drives APC maturation, ultimately priming adaptive immune responses [73]. In murine models of DED, conjunctival NK and natural killer T cells (NKT) produce IL-6 and IL-23, activating DCs and enhancing Th17 responses [74]. Notably, NK cell depletion reduces cytokine levels and preserves corneal integrity, underscoring a critical NK–DC–Th17 axis in early DED. Similarly to macrophages, this evidence supports the role of NK cells not only in sustaining local inflammation at the OS but also in contributing to the oversensitization of the adaptive immune system seen in DED.

## 7. Adaptive Immune System in DED

Once primed, the adaptive immune system plays a central role in the chronicity of DED. Autoreactive T cells drive sustained inflammation of the OS, in particular, CD4^+^ T helper subsets Th1 and Th17 cells. These mediate further cytokine-driven epithelial damage and perpetuate immune dysregulation and are unimpeded by impaired T regulatory cells [25].

The activation, migration, and interaction of APCs with naive T cells is central to the initiation of the adaptive immune response in DED and has been well established [27,75]. In murine models, the migration of APCs towards draining lymph nodes is facilitated by the upregulated expression of C-C chemokine receptor-7 (CCR7), which guides their exit from the OS to lymph nodes by responding to specific ligands CCL19 and CCL21, found in high density in corneal lymphatics and draining lymph nodes [76,77]. Within the lymph node microenvironment, APCs prime naive T cells by MHC–antigen interaction and costimulatory engagement (e.g., CD80/CD86 with CD28) towards CD4^+^ Th1 and Th17 effectors [78,79].

### 7.1. Th1 Cells

The effect of Th1 cells in DED was initially noted to be more pronounced in the acute phase, as evidenced by the presence of its related cytokine IFN-γ in murine models of early DED and its reduced prominence later in disease progression [80]. DED-primed Th1 cells upregulate chemokine receptors CCR5 and CXCR3, facilitating their targeted migration from the lymph nodes back to the inflamed OS via corresponding chemokine gradients, as demonstrated in murine models [30].

IFN-γ was initially thought to amplify its own production by upregulating IL-12 receptor expression, promoting further Th1 differentiation and inducing chemokines (CXCL9, CXCL10, CXCL11), which recruit and retain Th1 cells in inflamed tissues [81]. IFN-γ itself exerts its effects in DED by inducing GC loss and reducing mucin production, thus worsening tear film instability and perpetuating the vicious cycle of inflammation [82]. Moreover, loss of conjunctival GC likely disrupts local immune tolerance by impairing APC tolerance, leading to enhanced IL-12 production and pathogenic Th1/Th17 polarization as evidenced in murine models [83]. However, a review by Chen et al., scrutinizing the varied roles of Th-17 cells in DED, concluded that the exact source of IFN-γ in the acute phase of DED may be associated with NK cells, and its continued secretion throughout the course of the disease may be related to IFN-γ^+^IL-17^+^ “double-positive” Th17/1 cells [27].

### 7.2. Th17 Cells

The role of Th-17 cells in DED is significant and central to the pathogenesis of DED. Differentiation of Th-17 cells is largely dependent on the microenvironment and is initiated by the signal transducer and activator of transcription 3 (STAT3) signaling pathway after exposure to IL-6 and TGF-β secreted by APCs [84,85]. This is further regulated and promoted by the transcription factor retinoic acid–related orphan receptor gamma t (RORγt), which was found to be significantly upregulated in the OS tissues after exposure to desiccating stress in murine models [86]. Th17 cells then migrate back to the OS, specifically the conjunctiva, via chemokine receptors CCR6 and CCL20, where they undergo further differentiation following exposure to IL-1 and IL-23 [87].

A key cytokine produced by Th17 is IL-17, found in greater concentrations in tears of both non-SS and SS-DED patients, which stimulates MMP production and causes damage to the corneal epithelium [81,88]. However, there are several other key Th-17 subsets, including IL-10 producing Th17 cells, Th17/Th1 cells which co-produce IL-17 and IFN gamma, Th-17 producing Granulocyte-macrophage colony-stimulating factor (Th17GM-CSF), and more recently interleukin 17 receptor E (IL-17RE) and CCR10 producing Th17 cells in murine models [31,89,90,91].

Whilst IL-10 producing Th-17 cells may potentially play a regulatory role in DED (as seen in murine models), other subsets are more detrimental [89]. Co-producing IL-17 and IFNγ Th-17 cells are significantly pathogenic and drive epithelial apoptosis, lymphangiogenesis, APC maturation and potentially IFNγ production seen in chronic DED [27,89,90].

Granulocyte-macrophage colony-stimulating factor (GM-CSF) exerts its role in DED through stimulation of monocytic cells to produce proinflammatory cytokines such as IL-1β, IL-6, and IL-23. IL-6 and IL-23 further perpetuate Th17 differentiation [31]. In a murine model of DED, the IL-17RE^highCCR10^+^ Th17 subset exhibited enhanced JNK and p38 MAPK signaling. This is likely mediated through IL-17C/IL-17RE interaction, which reinforces and perpetuates their Th17 phenotype by sustaining IL-17A expression in vitro [91]. The presence of multiple subsets of Th-17 cells and their more hybrid phenotypes such as Th17/Th1 and CCR10 expression (typically found on T helper 22 cells) may suggest plasticity of Th17 cells which can transdifferentiate depending on the microenvironment as seen in murine models [91].

Alam et al. identified γδ T cells as a significant source of IL-17 in RXRα-deficient Pinkie mouse models, with elevated IL-17A and IL-17F expression that exacerbated DED [92]. They also demonstrated that 9-cis retinoic acid, the natural ligand for RXRα, suppresses IL-17 production from both γδ T cells and monocytes in vitro, suggesting that RXRα may act as a negative regulator of IL-17-driven inflammation [92].

### 7.3. Memory T Cells

Chronic DED is predominantly driven by a persistent Th17 response, particularly by effector memory Th17 cells [80]. Chen et al. used murine models to demonstrate that OS inflammation persists despite the absence of desiccating stress and is largely driven by continued IL-17 secretion from this population of cells [62]. Th17 memory cells also continue to promote further migration of effector T cells from lymph nodes (LN) to the OS via pathological lymphangiogenesis [80]. The same group also identified in pre-clinical models that IL-7 and IL-15 are critical for the maintenance of Th17 memory cells and promote continued survival via STAT5 and phosphoinositide 3-kinase–Akt (PI3K-Akt) pathways [93]. Moreover, IL-23 was found to promote the transition of Th17 effector cells into memory cells whilst IL-2 had an inhibiting effect on this pathway as demonstrated in murine models [94].

### 7.4. Tregs

Several studies examined Treg cells in DED, reporting various results. CD25^+^CD4^+^Foxp3^+^ Tregs are peripheral Tregs with important mechanisms of action, including: (1) granzyme-B and perforin mediated cytolysis, (2) the release of immunosuppressive cytokines including IL-10, TGF-β and IL-35, (3) the inhibition of DC maturation and function via the transendocytosis of co-stimulatory molecules (CD80/86) and (4) suppression via metabolic competition [37,95,96]. In DED murine models, their reduction led to increased severity of SS-like DED [97]. Conversely, the transfer of in vitro expanded Tregs into two different strains of DED murine models, BALB/c and C57BL/6, showed suppression of pathogenic CD4^+^ effector T cell-mediated inflammation [98].

In contrast, other reports demonstrated that Treg levels may remain unchanged in DED, but their suppressive function is compromised. Chauhan et al. noted preserved Treg numbers but impaired ability to restrain Th17 cells, likely due to IL-17A secretion by effector cells [99]. Similarly, the proinflammatory IL-6–rich microenvironment of DED inhibits Treg differentiation [100].

In patients with SS-associated DED, conjunctival biopsies showed marginally increased Treg counts without correlation to clinical severity, further suggesting that dysfunction rather than numerical deficit drives pathology [101]. This was further evidenced in stromal interaction molecule 1/2 (STIM1/2) Foxp3^+^ mice, where targeted deletion of calcium-sensing proteins STIM1/2 in Foxp3^+^ Tregs resulted in a fulminant SS-like phenotype characterized by lacrimal gland inflammation, lymphocytic infiltration, and IFN-γ–dominated transcriptional signatures [102]. Moreover, transcriptomic analyses revealed downregulation of core memory Treg genes in both mouse and human SS, suggesting that functional Treg impairment, not mere reduction, is a conserved and critical step in disease development [102].

The apparent discrepancies in Treg data across DED studies likely reflect differences in disease models, tissue compartments assessed, and methodological approaches to Treg identification, as well as the dynamic nature of Treg function across disease stages. This highlights the need for standardized methods to assess both Treg frequency and suppressive capacity in order to clarify their role in human DED pathogenesis.

### 7.5. B Cells and Humoral Dysfunction

B cells contribute to immune dysregulation in DED, but their role differs markedly between SS-associated DED and non-SS forms. In primary SS-associated DED, B cells are a central driver of pathology. Historically, glandular infiltrates were considered to be primarily CD4^+^ T cell–mediated, but recent studies show that B cells play a dominant role across both early and late disease stages [103,104]. In murine models such as the NOD.H-2h4 strain, B cells are the predominant immune infiltrates within lacrimal glands, with T cells providing additional contributions to the inflammatory milieu [105].

Within the salivary and lacrimal glands of SS patients, periductal lymphocytic infiltrates and ectopic germinal centers are often B-cell-dominated. Their hyperactivity is sustained by chemokines such as CXCL12, CXCL13, CCL19, and CCL21, as well as cytokine axes involving BAFF, IL-6, and IL-21 [106]. This dysregulated signaling drives clonal expansion, the production of autoantibodies (SSA/Ro, SSB/La, rheumatoid factor), and, in severe cases, malignant transformation into B-cell lymphomas. Beyond antibody secretion, B cells also function as APC and cytokine producers (IL-2, IL-12, TNF-α, IFN-γ), amplifying autoreactive T-cell responses and promoting tissue destruction [107]. Animal studies further support their pathogenic role, as passive transfer of autoantibodies alone can induce disease features [66].

By contrast, evidence for B-cell involvement in non-SS DED is far more limited. While B cells and plasma cells are detectable in the conjunctiva and OS tissues, their proportions do not differ significantly between non-SS DED patients and healthy controls [108]. Some data suggest that autoantibodies, such as those against kallikrein-13, can induce complement-dependent OS inflammation in murine DED models, pointing to a potential but secondary role for B-cell–mediated autoimmunity [66]. Activated B cells may also contribute indirectly by producing proinflammatory cytokines such as IL-6 and TNF-α [107]. However, unlike SS-associated DED, ectopic germinal center formation within lacrimal glands is rare in non-SS disease, underscoring the limited role of B cells in its pathogenesis.

In summary, B-cell dysregulation is a hallmark of SS-associated DED, driving autoantibody production, cytokine amplification, and tissue damage. In non-SS forms however, B cells appear to play a minor, context-dependent role, with disease pathogenesis being more strongly linked to T-cell–mediated mechanisms.

## 8. Neuroimmune Mediated Inflammation in DED

Neuroimmune dysregulation significantly contributes to the pathogenesis of DED by aberrantly modulating both innate and adaptive immune responses. As proposed in a review by Huang et al., this may be due to a bidirectional feedback loop in which proinflammatory cytokines released by immune cells damage peripheral nerves, triggering excessive neuropeptide release [109]. These neuropeptides, in turn, further activate immune pathways, amplifying OS inflammation and perpetuating chronic disease [109]. Yu et al. have underscored the role of the neuropeptide substance P (SP) from sensory nerve endings in enhancing MHC-II expression on DCs, thereby promoting subsequent T cell priming [75]. Moreover, the SP–neurokinin 1 receptor (NK1R) axis directly acts on pathogenic Th17GM-CSF cells, significantly increasing GM-CSF production and exacerbating dry eye disease severity in murine models [110]. This highlights a novel neuroimmune pathway through which neuropeptides enhance effector T cell activity and OS inflammation [110].

In addition, SP/NK1R signaling has been implicated in pathological lymphangiogenesis in DED, where it upregulates Vascular Endothelial Growth Factor (VEGF)-C, VEGF-D, and VEGFR-3 expression, promoting lymphatic vessel growth and facilitating APC trafficking to draining lymph nodes [111]. Moreover, whilst Tregs expressing NK1R are found in increased quantities in DED, their abnormal expression of critical immunomodulatory markers CTLA-4, PD-1, TGF-β, and IL-10 demonstrates an impaired suppressive capacity against effector T cells, suggesting a compromised regulatory phenotype [112,113]. SP may also be implicated in the promotion and maintenance of memory Th17 cells [80]. In vitro studies have shown increased conversion of effector T cells to memory Th17 cells when cultured with SP [80]. Further, when cultured with Th17 memory cells, SP continued to preserve the cells [114].

Calcitonin gene-related peptide (CGRP) plays a potentially more complex and unclear role in DED, exhibiting both immunosuppressive and proinflammatory roles depending on the microenvironment [109,115]. Its immunosuppressive activity includes the inhibition of APCs by Langerhans cells and attenuation of contact hypersensitivity through the suppression of mast cell-derived tumor necrosis factor [116]. However, clinical data on CGRP and SP remain conflicting. Some studies report reduced tear concentrations of CGRP and SP, particularly in severe or chronic DED, where corneal nerve loss may impair neuropeptide production and secretion, thereby diminishing their homeostatic and immunomodulatory functions [115,116,117]. Conversely, other studies demonstrate increased neuropeptide levels, particularly in DED subtypes with prominent neuroinflammation or post-surgical neuropathic pain, suggesting upregulation in response to inflammatory stimuli or nerve sensitization [112,118]. These discrepancies possibly reflect underlying disease heterogeneity, variation in nerve integrity, and/or stage-specific dynamics (early inflammatory vs. late neurodegenerative disease).

Experimental models further underscore the complexity of neuroimmune interactions. In murine studies, tear hyperosmolarity alone disrupted neuroimmune homeostasis via TRPV1, NF-κB activation in conjunctival epithelium, leading to DC maturation, memory CD4^+^ T cell priming, corneal nerve loss, and impaired mucosal tolerance [119]. Adoptive transfer of these T cells induced DED in naive mice, establishing hyperosmolarity as a direct pathogenic trigger [119]. Complementing these findings, studies in guinea pig models of aqueous tear deficiency demonstrate that chronic dryness also sensitizes TRPV1-expressing corneal nociceptors, enhancing blink reflexes and neuronal calcium responses to capsaicin [120]. This neuroplasticity likely contributes to the ocular hyperalgesia and discomfort characteristic of DED [120].

## 9. Tear Biomarkers of DED

The pathophysiology of dry eye disease involves a complex interplay of immune, epithelial, and neuronal factors at the OS. Numerous biomarkers have been identified that reflect distinct aspects of disease activity, including inflammatory mediators, chemokines, cytokines, matrix metalloproteinases, and regulatory molecules (Table 1). They play roles in promoting or modulating OS inflammation, epithelial barrier disruption, and immune cell recruitment.

## 10. Therapeutic Implications

While the initiating events in DED remain unclear, sustained immune dysregulation, marked by aberrant activation of innate and adaptive immune pathways, plays a central role in perpetuating OS inflammation. This understanding has driven a shift from symptomatic treatments to targeted immunomodulation, aimed at disrupting specific molecular mediators of inflammation and restoring immune homeostasis. Despite advances in targeted immunotherapies, the mainstay of DED management remains tear supplementation. Sodium hyaluronate, particularly when combined with pranoprofen, alleviates hyperosmolar stress on the OS but does not directly modulate immunity [121]. Diquafosol, recommended in the TFOS DEWS III report, acts primarily by stimulating fluid and mucin secretion; however, proteomic analyses suggest it may also exert indirect immunoregulatory effects by stabilizing the tear film and thereby supporting OS immune homeostasis [121,122,123].

Several currently approved agents exert immunomodulatory effects, albeit with variable efficacy [124]. Cyclosporine A, a topical calcineurin inhibitor, suppresses IL-2–mediated T cell activation, reduces epithelial apoptosis, and has been shown to restore GC density in DED. Lifitegrast, a lymphocyte function-associated antigen-1 (LFA-1) antagonist, blocks T cell adhesion and migration through competitive inhibition of the LFA-1/intercellular adhesion molecule 1 (ICAM-1) interaction on the OS. Topical corticosteroids, though effective in rapidly reducing inflammatory mediators such as MMP-9, IL-1β, and TNF-α via suppression of NF-κB and activator protein 1 (AP-1), carry well-documented risks with prolonged use, including ocular hypertension, cataract formation, and susceptibility to infection [125]. Recently, Tacrolimus, a macrolide immunomodulator that inhibits T cell activation through calcineurin blockade, has been compared with Cyclosporine A in a randomized controlled trial and showed equally effective outcomes in reducing the need for artificial tear supplementation compared with placebo. No significant difference between treatments, however, has been observed in patients with severe DED secondary to SS [123].

Therapeutic advances have shifted focus toward precision immunomodulation targeting the upstream molecular and cellular drivers of innate immune dysregulation in DED. Modulating macrophage polarization is a potential therapeutic target, with murine studies demonstrating that shifting macrophages from a proinflammatory M1 phenotype to an anti-inflammatory M2 phenotype can contribute to a reduction in proinflammatory cells and an increase in anti-inflammatory factors. In a benzalkonium chloride (BAC)-induced murine model of DED, treatment with M2 macrophage–derived extracellular vesicles (M2-EVs) improved tear production, preserved corneal integrity, and downregulated inflammatory cytokines [126]. Zhou et al. highlighted the α7 nicotinic acetylcholine receptor (α7nAChR) as a regulator of macrophage-driven inflammation; activation of this receptor reduced OS inflammation via neuroimmune crosstalk in DED murine models [127].

Endogenous counter-regulatory molecules such as pigment epithelium-derived factor (PEDF) have also demonstrated immunosuppressive effects [128]. In both murine and human cells, elevated PEDF expression in the corneal epithelium and tear film suppressed key proinflammatory cytokines, including IL-1β, IL-6, TNF-α, and IL-17A, and reduced Th17 cell density through inhibition of the p38 MAPK and JNK pathways [128]. A ROS-responsive microneedle patch (CE-MN), loaded with cyclosporin A and the antioxidant epigallocatechin gallate (EGCG), enabled sustained periocular delivery to the lacrimal gland. In an SS-DED model, CE-MN suppressed macrophage activation and oxidative stress, while attenuating downstream Th1 and Th17-mediated inflammation more effectively than conventional eye drops [129].

Several potential strategies that target the adaptive immune response have emerged. A number of murine studies have shown that neutralizing IL-17A or blocking its upstream regulators improves OS quality. Local CCL20 neutralization reduces Th17 cell infiltration and inflammation in vitro and in vivo, while RXRα agonism with 9-cis retinoic acid attenuates Th17-driven inflammation and preserves goblet cells in the Pinkie mouse model [87,92]. Topical inhibition of phosphodiesterase type-4 (PDE4) with Cilomilast significantly suppressed IL-17 and IL-23 expression in conjunctival tissue and draining lymph nodes, reduced CD11b^+^ APC infiltration, and downregulated IL-1β, IL-6, and TNF-α [130]. This improved clinical outcomes, with therapeutic efficacy comparable or superior to both dexamethasone and cyclosporine in DED murine models [130]. However, when Secukinumab (a human monoclonal antibody that neutralizes IL-17A) was utilized systemically in patients with DED, it showed no significant amelioration of DED symptoms [131]. A phase II clinical trial focusing on the topical administration of an IL-17A antagonist has yet to release its results [132]. The apparent disparity between the promising pre-clinical data and clinical trial results of IL-17A neutralization further emphasizes the importance of delivery route, target engagement in the relevant tissue, and endpoint selection in translating cytokine-targeted therapies.

In chronic DED, targeting IL-7 and IL-15 with topical anti-IL-7 and anti-IL-15 antibody treatments in vivo murine models showed amelioration of disease severity; however, this could not target memory Th17 cells in draining lymph nodes [93]. Moreover, a murine study employing CCL22-releasing microspheres demonstrated increased recruitment of endogenous Tregs to the lacrimal gland, leading to improved epithelial integrity, reduced IFN-γ–mediated inflammation, and a restored Treg:effector T cell ratio [133].

Targeting neuroimmune signaling in DED is another therapeutic avenue that has been explored in the last decade. Topical blockade of NK1R using antagonists such as spantide and CP-99,994 significantly suppressed Th17 responses, reduced corneal lymphangiogenesis, and improved clinical outcomes [75,111]. Pyroptosis, a form of proinflammatory programmed cell death, and its associated polo-like kinase 1–cell division cycle 25C–cyclin-dependent kinase 1 (Plk1–Cdc25c–Cdk1) axis, can also be targeted using CP-99,994. This has led to reduced IL-6, IL-1β, and TNF-α levels, and further ameliorated OS inflammation in the murine DED model [134].

More recently, mesenchymal stem cell (MSC) therapy has emerged as a promising immunomodulatory strategy for the treatment of DED. MSCs derived from bone marrow, adipose tissue, and umbilical cord modulate ocular inflammation through both cell-mediated and paracrine mechanisms [135]. In a murine T cell–driven model of DED, local infusion of human or mouse MSCs suppressed CD4^+^ T cell proliferation and IFN-γ production, reducing OS inflammation and restoring GC density and tear secretion. These effects occurred independently of Treg induction or indoleamine 2,3-dioxygenase (IDO)-mediated tryptophan metabolism, suggesting alternative regulatory pathways, including transient recruitment of other immunosuppressive cells or species-specific mechanisms such as inducible nitric oxide synthase in murine MSCs [136]. Similarly, in a murine SS-DED model, bone marrow–derived mouse MSCs improved lacrimal gland secretory function and increased aquaporin 5 expression, while reducing lymphocytic foci size without significant changes in Foxp3^+^ Tregs or stromal cell-derived factor 1 (SDF-1)/CXCR4 signaling [137].

MSC-derived extracellular vesicles (MSC-EVs) demonstrate comparable efficacy: umbilical cord MSC-EVs downregulated the IRAK1/TAB2/NF-κB pathway via miRNAs, including miR-125b and let-7b, suppressing proinflammatory cytokines in murine DED models [138]. Adipose-derived MSC exosomes (mADSC-Exos) attenuated hyperosmotic stress–induced inflammation by reducing IL-1β, IL-6, and NOD-, LRR- and Pyrin domain-containing protein 3 (NLRP3) inflammasome activation in murine models, while promoting tear film stability and epithelial repair [139]. Additionally, human umbilical cord MSC–derived exosomal microRNA-146a (miR-146a) suppressed apoptosis and inflammation in hyperosmotic-stressed human corneal epithelial cells and a murine DED model by upregulating sequestosome 1 (SQSTM1), revealing a novel miRNA-mediated protective axis [140].

## 11. Conclusions

Our understanding of DED has shifted from a purely symptomatic perspective to one that recognizes dysregulated innate, adaptive, and neuroimmune pathways as central drivers of OS inflammation, highlighting the pivotal role of immune mechanisms in disease perpetuation and progression.

## 12. Future Directions

Advances in molecular and translational research have identified promising targeted immunomodulatory strategies such as Th17 inhibition, macrophage modulation, neuropeptide receptor blockade, and stem cell therapies. However, clinical translation remains limited by disease heterogeneity, lack of robust biomarkers, and incomplete understanding of human ocular immunopathology. Preclinical models, particularly murine systems, have provided critical mechanistic insight into immune dysregulation, yet their relevance to human disease is constrained, as evidenced by differences in outcomes between animal models and clinical trials. Moreover, studying large human populations is challenging due to variability in disease severity, phenotype, and access to standardized immunological assays. Future research should therefore prioritize precise immunophenotyping to enable personalized treatment and the development of reliable biomarkers for diagnosis and monitoring. Moreover, well-designed clinical trials exploring novel topical and cell-free immunotherapies are needed to bridge mechanistic insights from preclinical studies to human DED in order to restore immune homeostasis and improve patient outcomes.

## Figures and Tables

**Table 1:** Up (↑) and downregulated (↓) biomarkers in DED and their role in disease activity.

Category	Biomarker	Response in DED	Functional/Pathophysiological Role	Ref.
Chemokines	CCL2	↑	Drives basal epithelial cells to act as ‘non-professional APCs’ in further activating immune response	[53]
	CCL20	↑	Aids migration of Th17 cells back to OS, specifically the conjunctiva	[87]
	CCL5	↑	Promotes T cell recruitment	[6,37]
	CXCL10	↑	Recruits Th1 cells to the OS through CXCR3 signaling, amplifying local inflammation	[6,37]
	CXCL9	↑	Activates T cells and sustains chronic inflammatory responses via CXCR3	[6,37]
	CXCL1	↑	Activates TRPV1 and ADAM17 which contribute to ocular pain and epithelial barrier disruption	[71]
Cytokines	ACPA	↑	Generated during neutrophil NETosis, induces OS inflammation in murine models	[70]
	GM-CSF	↑	Stimulates monocytic cells to produce proinflammatory cytokines such as IL-1β, IL-6, and IL-23 with IL-6 and IL-23 further perpetuating Th17 differentiation	[31]
	IFN-γ	↑ in early DED ↓ in later disease progression	Induces epithelial damage and disrupts homeostasis of OS NK activation promotes IFN- γ-mediated inflammation and drives APC maturation which primes adaptive immune response. Induces GC loss and reduces mucin production	[40,47,50]
	IL-1	↑	Allows Th17 cells to undergo further differentiation at the conjunctiva.	[87]
	IL-10	↓	Exacerbates goblet cell loss and Th17-mediated pathology contribute to impaired suppressive capacity against effector T cells.	[39,78,79]
	IL-12	↑	Leads to further Th1 polarization	[83]
	IL-15	↑	Maintains Th17 memory cells and promotes continued survival	[93]
	IL-17	↑	Disrupts corneal epithelium barrier integrity, stimulates MMP production, and promotes inflammation and apoptosis	[81,88]
	IL-17C	↑	Enhances JNK and p38 MAPK signaling though IL-17C/IL17RE interaction therefore reinforces and perpetuates Th17 phenotype	[91]
	IL-1β	↑	Promotes epithelial damage, upregulates proinflammatory mediators, and enhances immune cell activation	[27,50,52]
	IL-2	-	Inhibits differentiation of Th17 effector cells into memory cells	[94]
	IL-20	↑	Promotes macrophage recruitment and increases inflammatory signaling in OS	[29]
	IL-23	↑	Allows Th17 cells to undergo further differentiation at the conjunctiva and promotes transition into memory cells	[6,13,41,55,64]
	IL-6	↑	Activates DCs and enhances Th17 responses; Initiates Th-17 cell differentiation via STAT3 signaling pathways; Exhibits inhibitory effect on Treg differentiation.	[6,13,41,52,53]
	IL-7	↑	Helps maintain Th17 memory cells and promotes continued survival	[93]
	IL17A	↑	Promotes neutrophil recruitment, epithelial barrier disruption, and proinflammatory cytokine production at OS	[61,70]
	IL17F	↑	Stimulates epithelial cells and immune cells to release inflammatory mediators and chemokines	[92]
	TGF-β	↑	Induces Th17 cells and contributes to impaired suppressive capacity against effector T cells.	[6,37]
	TNF-β	↑	Initiates Th-17 cell differentiation via STAT3 signaling pathways.	[84,85]
	TNF-α	↑	upregulates proinflammatory cytokines, disrupting epithelial barrier integrity, and amplifying immune cell infiltration	[27,50,52]
Chemokine receptors	CCR6	↑	Aids migration of Th17 cells back to OS, specifically the conjunctiva	[87]
	CXCR3	↑	Facilitates migration of DED-primed Th1 cells from lymph nodes back to inflamed OS	[30]
Damage-Associated Molecules (DAMPS)	HMGB1	↑	Activates TLR pathways and induces proinflammatory cytokine and MMP-9 release	[55]
	HSP-60	↑	Activates TLR pathways, leading to cytokine release and immune cell recruitment	[55]
Enzymes/receptors related to pain and epithelial integrity	ADAM17	↑	Contributes to ocular pain and epithelial barrier disruption	[71]
	TRPV1	↑	Contributes to ocular pain and epithelial barrier disruption	[71]
Growth/Neuroimmune factors	CGRP	↑ and ↓	Exhibits both an immunosuppressive and proinflammatory role depending on the microenvironment; Inhibits APCs through suppression of mast cell-derived TNF; upregulated in response to inflammatory stimulation or nerve sensitization.	[75,81,82,85]
	VEGF, VEGF-D, VEGFR-3	↑	Promotes lymphatic vessel growth and facilitates APC trafficking to draining lymph nodes.	[111]
Immune checkpoint molecules	CTLA-4	↑	Impairs suppressive capacity against effector T cells	[112,113]
	PD-1	↑	Impairs suppressive capacity against effector T cells	[112,113]
Matrix-degrading enzymes	MMP-9	↑	Degrades epithelial basement membrane components and disrupts tight junction proteins	[27,50,52]
Signaling molecules	NF-κB	↑	Drives early upregulation of proinflammatory cytokines, promoting immune cell activation	[27,29]
	NFAT5	↑	Promotes early cytokine upregulation and immune cell activation	[27,29]
Toll-Like Receptors	TLR4	ITLR mRNA ↑ TLR protein levels ↓	Recognizes DAMPs (like HMGB1) and microbial products, activating NF-κB and driving cytokine/chemokine release	[46]
	TLR9	TLR9 mRNA ↓ TLR9 protein ↓	Impairs local immune regulatory function at OS	[54]
Transcription factors	RORγt	↑	Regulates and promotes Th-17 cell differentiation	[86]
	RXRa	↓	Exacerbates goblet cell loss and Th17-mediated pathology.	[72]

## Data Availability

No new data were created or analyzed in this study. Data sharing is not applicable to this article.

## Abbreviations

ACPA	Anti-citrullinated protein autoantibodies
ADAM17	A Disintegrin And Metalloproteinase 17
ADDE	Aqueous-deficient dry eye
AP-1	Activator Protein 1
α7nAChR	α7 Nicotinic Acetylcholine Receptor
BAC	Benzalkonium Chloride
BALB/c	BALB/c mouse strain
CCL5	C-C motif chemokine ligand 5
CCR7	C-C chemokine receptor 7
CGRP	Calcitonin Gene-Related Peptide
CE-MN	Reactive Oxygen Species (ROS)-responsive Microneedle Patch
CP-99,994	N1KR antagonist
CXCL9	C-X-C motif chemokine ligand 9
DC	Dendritic Cell
DAMPs	Danger Associated Molecular Patterns
EDE	Evaporative Dry Eye
EGCG	Epigallocatechin Gallate
FoxP3	Forkhead Box Protein P3
HMGB1	High Mobility Group Box 1
HSP-60	Heat Shock Protein 60
ICAM-1	Intercellular Adhesion Molecule 1
IDO	Indoleamine 2,3-dioxygenase
IL	Interleukin
IL-17C	Interleukin 17C
IL-17RE	Interleukin 17 Receptor E
JNK1/2	c-Jun N-terminal kinase 1/2
LFA-1	Lymphocyte Function-Associated Antigen-1
LN	Lymph Nodes
MAPKs	Mitogen-Activated Protein Kinases
M1	Classically activated (proinflammatory) macrophage
M2	Alternatively activated (anti-inflammatory) macrophage
M2-EVs	M2 Macrophage–Derived Extracellular Vesicles
MD2	Myeloid Differentiation Factor 2
MHC	Major Histocompatibility Complex
miR-146a	MicroRNA-146a
MSC	Mesenchymal Stem Cell
MSC-EVs	MSC-Derived Extracellular Vesicles
mADSC-Exos	Adipose-Derived Mesenchymal Stem Cell Exosomes
mRNA	Messenger Ribonucleic Acid
NF-κB	Nuclear Factor kappa-light-chain-enhancer of activated B cells
NFAT5	Nuclear Factor of Activated T Cells 5
NK	Natural Killer Cells
NKT	Natural Killer T Cells
NETs	Neutrophil Extracellular Traps
NLRP3	NOD-, LRR- and Pyrin Domain-Containing Protein 3
PAD4	Peptidylarginine Deiminase 4
PEDF	Pigment Epithelium-Derived Factor
PI3K-Akt	Phosphoinositide 3-Kinase–Akt
Plk1–Cdc25c–Cdk1	Polo-Like Kinase 1—Cell Division Cycle 25C—Cyclin-Dependent Kinase 1
PDE4	Phosphodiesterase Type-4
PD-L1	Programmed Death Ligand 1
RASγt	Retinoic Acid–Related Orphan Receptor Gamma t
RXRα	Retinoid X Receptor Alpha
ROS	Reactive Oxygen Species
RORγt	Retinoic Acid–Related Orphan Receptor Gamma t
SDF-1	Stromal Cell-Derived Factor 1
SP	Substance P
STIM1/2	Stromal Interaction Molecule 1/2
SQSTM1	Sequestosome 1
STAT3	Signal Transducer and Activator of Transcription 3
Th17GM-CSF	Th-17 Producing Granulocyte-Macrophage Colony-Stimulating Factor
TGF	Tumor Growth Factor
Tregs	T Regulatory Cells
TRPV1	Transient Receptor Potential Vanilloid 1
ZO-1	Zonula Occludens-1
